# Microarray Analyses of Genes Differentially Expressed by Diet (Black Beans and Soy Flour) during Azoxymethane-Induced Colon Carcinogenesis in Rats

**DOI:** 10.1155/2012/351796

**Published:** 2012-02-08

**Authors:** Elizabeth A. Rondini, Maurice R. Bennink

**Affiliations:** Department of Food Science and Human Nutrition, 106 GM Trout Building, Michigan State University, East Lansing, MI 48824, USA

## Abstract

We previously demonstrated that black bean (BB) and soy flour (SF)-based diets inhibit azoxymethane (AOM)-induced colon cancer. The objective of this study was to identify genes altered by carcinogen treatment in normal-appearing colonic mucosa and those attenuated by bean feeding. Ninety-five male F344 rats were fed control (AIN) diets upon arrival. At 4 and 5 weeks, rats were injected with AOM (15 mg/kg) or saline and one week later administered an AIN, BB-, or SF-based diet. Rats were sacrificed after 31 weeks, and microarrays were conducted on RNA isolated from the distal colonic mucosa. AOM treatment induced a number of genes involved in immunity, including several MHC II-associated antigens and innate defense genes (*RatNP-3, Lyz2, Pla2g2a*). BB- and SF-fed rats exhibited a higher expression of genes involved in energy metabolism and water and sodium absorption and lower expression of innate (*RatNP-3, Pla2g2a, Tlr4, Dmbt1*) and cell cycle-associated (*Cdc2, Ccnb1, Top2a*) genes. Genes involved in the extracellular matrix (*Col1a1, Fn1*) and innate immunity (*RatNP-3, Pla2g2a*) were induced by AOM in all diets, but to a lower extent in bean-fed animals. This profile suggests beans inhibit colon carcinogenesis by modulating cellular kinetics and reducing inflammation, potentially by preserving mucosal barrier function.

## 1. Introduction

Colorectal cancer (CRC) is one of the most common neoplasms afflicting industrialized societies [1]. In 2008, there were 609,051 deaths due to colorectal cancer worldwide, with 50,640 cases in the United States alone [1]. Both genetic and environmental exposures have been implicated in the etiology of CRC, and it has been estimated that up to 75% of cases may be preventable by adequate diets and regular exercise [2–4]. Consumption of diets low in red meat and alcohol and high in vegetables and cereal grains is generally associated with a decreased risk of developing CRC [4–6]. Additionally, populations consuming higher intakes of legumes (peas, beans, lentils, peanuts) are reported to have a lower risk of [6–12] and mortality from CRC [13].

It has long been known that dietary patterns modulate the incidence and mortality of colorectal cancer [2, 3], however, identification of specific mechanisms has been limited. The azoxymethane- (AOM-) induced colon cancer model in rodents has been utilized extensively to examine dietary influences on colon cancer. Tumors develop almost exclusively in the colon, primarily in the distal region, similar to the distribution observed in humans from high-risk areas. Additionally, many of the common genetic and pathogenic changes contributing to human colon cancers are also observed during AOM-induced colon carcinogenesis [14–19]. Although *Apc* mutations are infrequently detected [20], mutations in GSK-3*β* phosphorylation consensus sites on *β*-catenin are present in up to 77% of AOM-induced colon cancers as well as in early preneoplastic lesions [17, 21–23]. These sites are important for downregulation of *β*-catenin by ubiquitination and result in stabilization and nuclear localization of the protein [23]. Activating mutations on codon 12 and 13 of the k-*ras* gene [14, 15], upregulation of cyclo-oxygenase 2 (COX-2), and inducible nitric oxide synthase (iNOS), as well as alterations in transforming growth factor *β* signaling, are also common features to both human and AOM-induced colon cancers [24–29]. Using this model, experiments conducted in our laboratory [30–33] and by others [34–36] have demonstrated the potential of bean-based diets to inhibit AOM-induced colon cancer. For example, Hughes et al. [35] and Hangen and Bennink [32] found that rats fed dry beans (pinto, navy, or black beans) had a 50–57% lower incidence of colon cancer than rats fed a casein-based diet. Similarly, in a series of experiments, Bennink et al. [30, 31, 33] reported a significant reduction in colon tumor incidence and tumor burden in rats fed defatted soy flour compared to casein-fed control animals.

The purpose of the current investigation was to elucidate cellular mechanisms underlying colon cancer inhibition by beans *in vivo*. Microarrays were performed on mRNA isolated from distal colonic epithelium of saline and AOM-injected F344 rats fed either a casein (AIN), black bean (BB), or defatted soy flour (SF) diet for 31 weeks. We chose to focus on the distal segment because most tumors develop in this area using standard protocol (15 mg/kg AOM) and there is evidence for site-specific effects of food constituents on tumorigenesis [37]. It was anticipated that genes most important to dietary suppression of colon cancer would be similarly affected by black beans and soy flour and have altered expression (increased or decreased) that corroborated tumor incidence. The profile of genes altered in this experiment suggests aberrant activation of innate and adaptive immune responses are permissive for colon carcinogenesis, whereas inhibition of tumor promotion by bean feeding is associated with modulation of genes involved in crypt cell homeostasis, innate defense, and extracellular matrix components.

## 2. Materials and Methods

### 2.1. Animal Care and Experimental Diets

This study was conducted in conformity with the regulatory guidelines of the Michigan State University Institutional Animal Care and Use Committee. Ninety-five male Fischer (F344) rats were obtained from Harlan Sprague-Dawley (Indianapolis, IL) at 3 weeks of age and housed in plastic cages (2-3 rats/cage) in temperature-(23°C ± 2°) and humidity- (40–60%) controlled rooms with a 12-hour light/dark cycle. Throughout the experiment, animals had free access to food and distilled water and were assessed daily for health status and monthly for weight gain.

One control and two experimental diets were formulated based on the AIN-93G rodent diet with modifications [38], to contain the majority (i.e., >85%) of protein from either (1) high nitrogen casein (AIN), (2) black beans (BB), or (3) defatted soy flour (SF) (Archer Daniels Midland; Decatur, IL) and matched to have similar nutrient : energy ratios (Table 1). Black beans were soaked overnight in distilled water, cooked in a steam jacket kettle for 30 minutes, dried at 58°C, and then finely ground to pass through a 1.6 mm diameter screen prior to mixing with other diet ingredients. All diets contained approximately 18.9% (wt/wt) total protein, 11.3% dietary fiber, and 16.7% fat (wt/wt). Casein and tryptophan were added to black bean diet and methionine was added to all diets to increase the amino acid score to >90%. Lard, corn, and soybean oil were added to all diets, adjusted so the total saturated (SFA) : monounsaturated (MUFA) : polyunsaturated (PUFA) fatty acid ratios were 1.0 : 1.2 : 1.1, respectively.

### 2.2. Experimental Design

Upon arrival, animals were fed the control (AIN) diet and allowed one week to acclimatize to new conditions. At 4 and 5 weeks of age, rats received subcutaneous injections (100 *μ*L) of either 15 mg/kg of azoxymethane (AOM) prepared in saline (*n* = 75; Ash Stevens, Detroit, MI) or saline (saline, *n* = 20). Animals were fed the control (AIN) diet until one week after the second injection, when they were randomized by weight to either continue on the control (AIN) diet or to be fed one of the experimental diets (BB or SF). At 36 weeks of age, animals were sacrificed by CO_2_ inhalation and exsanguination, and the colon was immediately excised, opened longitudinally, and rinsed briefly in tap water to remove debris. Macroscopic tumors, when present, were excised and stored at −80°C to be analyzed as a separate part of this study. The colon was then transected into proximal and distal segments and epithelial cells were collected by gently scraping normal-appearing mucosa from the distal half of the colon (excluding the lowermost 1 cm) with a glass slide. Samples were snap frozen and stored at –80°C until RNA extraction could be performed.

### 2.3. Microarray Target Preparation and Hybridization

Affymetrix RU34A rat genome chips (Santa Clara, CA) were used in this experiment. For total RNA isolation, the distal colonic mucosa was homogenized using a Tekmar homogenizer in TRIzol reagent containing RNase-free glycogen according to the manufacturer's instructions (Gibco, Carlsbad, CA). After RNA extraction, samples were cleaned with RNeasy minicolumns (Qiagen, Valencia, CA), quantified using a UV spectrophotometer (*A*_260_/*A*_280_), and the quality of RNA assessed by agarose-formaldehyde gel electrophoresis. Only high quality RNA was used in subsequent steps.

Biotinylated cRNA was prepared in accordance with instructions supplied in the GeneChip Expression Manual (Affymetrix, Santa Clara, CA). Double-stranded cDNA was synthesized from 10 *μ*g of total RNA, pooled from 2–4 animals/treatment, using T7-(dT)_24_ primers containing a T7 RNA polymerase promoter site (Proligo, Boulder, CO) and the Superscript II system (Invitrogen, Carlsbad, CA). Biotinylated cRNA was prepared using the Enzo BioArray HighYield RNA Transcript Labeling Kit (Affymetrix, Santa Clara, CA) and then purified with RNeasy minicolumns. Approximately 15 *μ*g cRNA was fragmented at 94°C for 35 minutes and hybridized to RGU34A rat genome chips for 16 hours at 45°C. Following hybridization, arrays were washed and stained with a streptavidin-phycoerythrin conjugate on an Affymetrix Fluidics station according to standard protocol. Processed arrays were scanned at 570 nm using a Hewlett Packard GeneArray Scanner.

### 2.4. Confirmation of Gene Changes by Quantitative Reverse Transcriptase PCR (*q*RT-PCR)

Select genes were confirmed using *q*RT-PCR. Gene-specific primers for cell division cycle 2 (*Cdc2*), cyclin B1 (*Ccnb1*), topoisomerase II alpha (*Top2A*), group IIA secretory phospholipase A2 (*Pla2g2a*), fibronectin 1 (*Fn1*), collagen, type I, alpha 1 (*Col1a1*), rat neutrophil (NP) defensin 3 (*RatNP-3*), aquaporin 8 (*Aqp8*), and 3-hydroxy-3-methylglutaryl-Coenzyme A synthase 2 (*Hmgcs2*) were designed with the Primer Express 2.0 program (Applied Biosystems, Foster City, CA). *β*-actin was used as an internal control. The sequences of the primer pairs used are available in Supplementary Table  1 available at doi:10.1155/2012/351796. Single-stranded (ss) cDNA was synthesized from 2.5 *μ*g of total RNA using T7-(dT)_24_ primers (Proligo, Boulder, CO) and the Superscript II system (Invitrogen, Carlsbad, CA). Reverse transcription was performed in a thermocycler following the Superscript first strand synthesis protocol (Invitrogen, Carlsbad, California).

Quantitative determination of gene expression was performed with the ABI Prism7000 (Perkin Elmer Corp., Foster City, CA) using the SYBR Green Universal Master Mix (Applied Biosystems, Foster City, CA). The reaction mixture (25 *μ*L total volume) contained 20 ng ss cDNA, 12.5 *μ*L SYBR Green Universal Master mix, and 10.5 *μ*L of diluted primers (300–600 nM). The real-time cycle conditions were as follows: PCR initial activation step at 95°C for 15 min and a total of 40 cycles for melting (95°C, 15 s) and annealing/extension (60°C, 1 min). All assays were performed in duplicate, using 3-4 samples per group (representing 9–12 animals/group) and relative fold-changes were quantified by using the comparative CT (ΔΔCT) method (User bulletin number 2, Applied Biosystems, Foster City, CA).

### 2.5. Statistical Analyses

Data for weight gain, microarrays, and *q*RT-PCR were analyzed using the General Linear Models procedure of SAS (SAS Institute, Cary, NC, Version 7.0). When statistical differences were detected with the *F* statistic, individual comparisons were made using the least significant difference (LSD) method. Tumor incidence data were analyzed with a *χ* ^2^ test using the Proc Freq procedure in SAS. Prior to statistical analyses, fluorescence intensity data from microarrays were globally scaled to a target intensity of 500 in Affymetrix Microarray Suite, Version 5.0 to control within-chip variations. Globally scaled data were then imported into GeneSpring (Silicon Genetics, Inc., Redwood City, CA, Version 6.0) for normalization and filtering. All chips were normalized to the median intensity of a set of invariant genes whose expression across all conditions (injection type, diet) after global scaling showed less than a 30% coefficient of variation (CV). A filtering step excluded genes not considered “Present” or “Marginal” in at least 42% of the individual samples. An additional filtering step limited the genes further to those exhibiting greater than 1.3 or less than 0.7 fold-change difference between diets or injection type. Normalized expression values were exported then analyzed using the GLM procedure of SAS. When present, duplicate transcripts were averaged prior to statistical analysis.

Differentially expressed transcripts (*P* < 0.05) were broadly grouped into categories based on known gene ontologies and biological functions reported in the literature. Gene ontologies were retrieved using the Affymetrix NetAffix Analysis Center (http://www.affymetrix.com/analysis/index.affx) and DAVID 6.7 functional analysis tool [39, 40]. Data are presented as mean fold-change differences standardized to the AIN (control) group for diet-dependent differences or to the saline-injected group for injection-dependent changes.

## 3. Results

### 3.1. Weight Gain and Tumor Incidence

**Figure 1:**
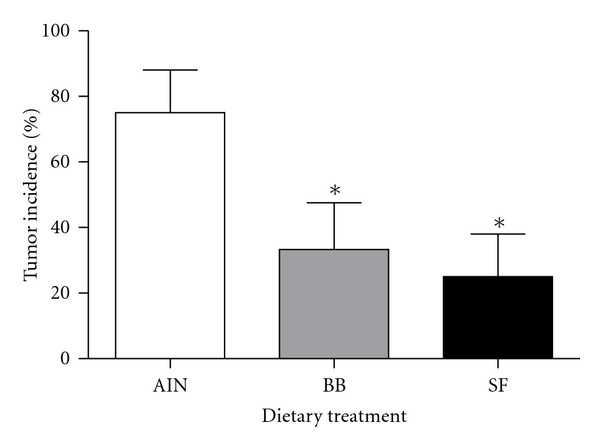
Tumor incidence in rats treated with the carcinogen azoxymethane and fed either a casein (AIN control), black bean- (BB-), or soy flour- (SF-) based diet. *Denotes significance compared to AIN controls (*P* < 0.05).

There were no significant effects of diet (AIN, BB, SF) or injection regime (AOM versus saline) on body weight gain. The total weight gain (g, LSM ± SEM) of rats while on experimental diets was AIN = 304 ± 7.4, BB = 287 ± 8.3, SF = 299 ± 8.2. There was a significant effect of diet on tumor incidence (**P** = 0.03). As previously established, bean-based diets inhibited tumor incidence by ~60% compared to rats fed the control diet (Figure 1).

### 3.2. Biological Classification of Gene Changes in Distal Colonic Mucosa during AOM-Induced Carcinogenesis

**Figure 2:**
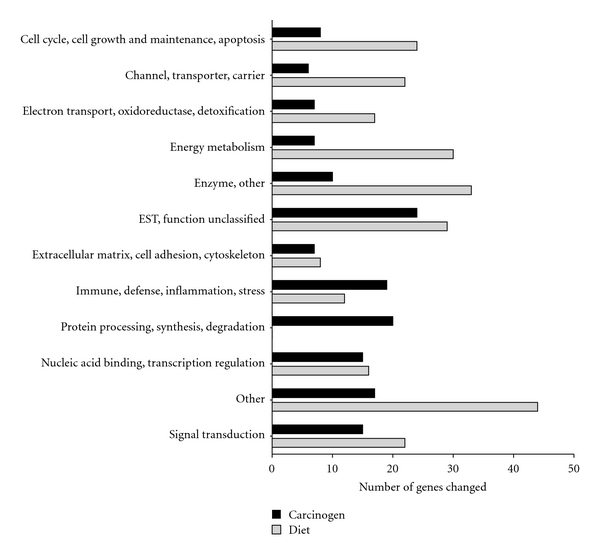
Functional classification of genes significantly altered by carcinogen (AOM) and by dietary treatment in the distal colon mucosa of rats detected by microarrays. A total of 155 genes were altered by carcinogen (AOM) and 257 by dietary treatment (AIN versus BB versus SF, *P* < 0.05).

Among the 8799 genes and ESTs present on the rat genome UG34A array, a total of 155 transcripts were significantly affected by injection regime (AOM versus saline), 257 by dietary treatment (AIN, BB, SF), and 5 were affected by both (*P* < 0.05). Transcripts differentially expressed by either carcinogen or diet were broadly classified into one of twelve functional categories and results are depicted in Figure 2.

### 3.3. Genes Differentially Expressed by Carcinogen (AOM) in Distal Colonic Mucosa

A total of 108 transcripts were higher and 47 lower in the colon of rats injected with carcinogen (AOM) compared to saline-injected controls (*P* < 0.05). As shown in Figure 2, a majority (55%) of transcripts affected were associated with immune, defense, inflammation (*n* = 19, 12%), signal transduction (*n* = 15, 10%), other (*n* = 17, 11%), or protein processing, synthesis, degradation (*n* = 20, 13%). Genes involved in antigen presentation (*RT1-Ba, RT1-Da, RT1-Bb, RT1-DMb, RT1-M3-1, RT1-Db1*, *CD74*), immune, defense, inflammation (*RatNP-3*, *Lyz2*, *Pla2g2a*), and components of the extracellular matrix (*Col1a1*, *Fn1, Col3a1*) were among those most highly induced by AOM-treatment (Table 2). Several of these genes have previously been shown to be overexpressed in carcinogen-induced colon cancer [41] and inflammatory conditions of the colon [42]. Several ribosomal proteins, including components of the 40S ribosomal protein subunit (*Rps7, Rps15, Rps17*, *Rps9, Rps4x*) and the 60S unit (*Rpl37*, *Rpl4, Rpl36a*) were also moderately induced by carcinogen compared to saline-injected controls.

### 3.4. Genes Differentially Affected by Dietary Treatment in Distal Colonic Mucosa

Compared to the AIN diet, feeding rats black beans (BB) significantly affected 188 genes (102 upregulated, 86 downregulated), and soy flour (SF) affected 140 genes (97 upregulated, 43 downregulated). Fifty three genes were significantly coinduced and 34 corepressed by BB and SF, representing 34% of gene changes, although an additional 24% showed the same direction of change. A majority of known transcripts affected by dietary treatment (68%) fell into one of five categories including other (*n* = 44, 17%), enzymes (*n* = 33, 13%), energy metabolism (*n* = 30, 12%), cell cycle, cell growth and maintenance, and apoptosis (*n* = 24, 9.3%), channel, transporter, carrier proteins (*n* = 22, 8.6%), and signal transduction (*n* = 22, 8.6%) see Figure 2.

A select listing of transcripts similarly affected the colon of bean-fed compared to casein-fed rats is presented in Table 3. As shown, bean-based diets coinduced a number of genes involved in fatty acid metabolism and gluconeogenesis (*Hmgcs2*, *Aldob*, *Pck1*, *Ech1*), electron transport, oxidoreductase, detoxification (*Cyp4b1*, *Gstm5*, *Gstm1*, *Cyp27a1*, *Prdx6*), and solute, ion transport (*Aqp8*, *Scnn1g*, *Slc12a7*, *Slc5a1*, *Slc16a1*). Among genes corepressed by beans included those involved in cell cycle (*Top2a*, *Ccnb1*, *Cdc2*), fatty acid desaturation (*Scd1*, *Scd*), extracellular matrix (*Col1a1*,* Fn1*), immune, defense, inflammation (*Dmbt1*, *Pla2g2a*, *RatNP-3*, *Tlr4, Ccxl14*), and nucleic acid binding, transcription regulation (*Egr1*, *Egr2*).

**Figure 3:**
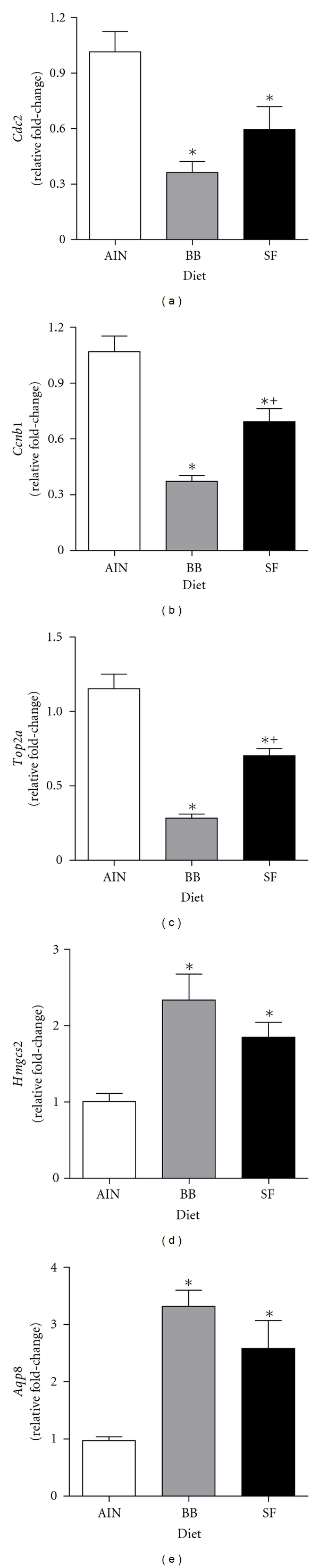
*q*RT-PCR analysis of genes for (a) *Cdc2*, (b) *Ccnb1*, (c) *Top2a*, (d) *Hmgcs2*, and (e) *Aqp8* in the distal colonic mucosa of rats fed either an AIN (control), black bean- (BB-), or soy flour- (SF-) based diet. Results were normalized to the housekeeping gene *β*-Actin and are presented as relative fold-changes (LSM ± SEM) standardized to the AIN (control) diet. *Denotes significance compared to AIN controls; ^+^denotes significance between BB and SF-fed animals (*P* < 0.05). *Abbreviations: Cdc2*, cell division cycle 2; *Ccnb1*, cyclin B1; *Top2a*, topoisomerase II alpha; *Hmgcs2, *3-hydroxy-3-methylglutaryl-Coenzyme A synthase 2; *Aqp8, *Aquaporin 8.

The relative expression of select genes (*Cdc2, Ccnb1, Top2a, Hmgcs2, *and* Aqp8*) was further evaluated by *q*RT-PCR. As shown in Figure 3, the mRNA for *Cdc2, Ccnb1, Top2a* were all significantly lower, whereas *Hmgcs2 *and* Aqp8 *were higher in the colon of BB- and SF-fed rats compared to controls (AIN *P* < 0.05). The direction of change was generally consistent with those obtained using microarrays (Table 3).

### 3.5. Genes Affected by Both Dietary Treatment and Carcinogen (AOM) in Distal Colonic Mucosa

**Figure 4:**
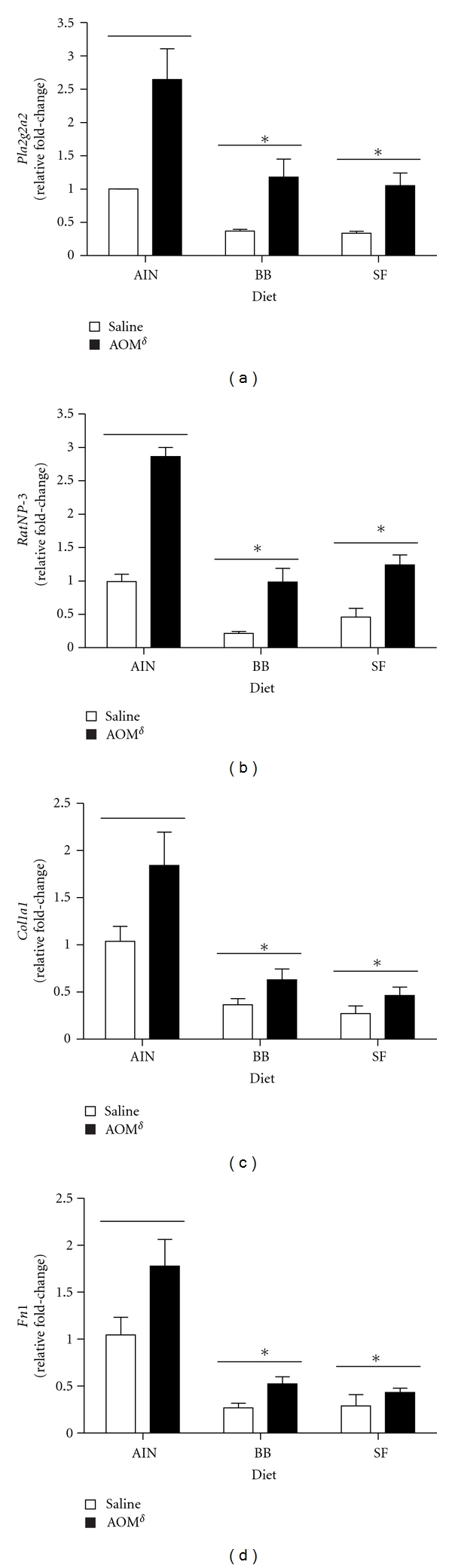
Relative fold-changes in (a) *Pla2g2a*, (b) *RatNP*-3, (c) *Col1a1*, and (d) *Fn1* detected with *q*RT-PCR. Results were normalized to the housekeeping gene *β*-Actin and are presented as mean fold-changes (LSM ± SEM) relative to the AIN(saline-injected) group. There were significant main effects for both diet and carcinogen for each gene presented (*P* < 0.05). *Denotes significant effect of diet compared to AIN controls; *δ* denotes significant effect of carcinogen treatment compared to saline controls (*P* < 0.05). Abbreviations: BB, black bean; SF, soy flour; *Pla2g2a*, phospholipase A2, group IIA; *RatNP*-3, rat neutrophil defensin 3; *Col1a1*, collagen, type I, alpha 1; *Fn1*, fibronectin 1.

Five genes were found to be influenced both by diet and carcinogen and are presented in Table 4. Transcripts for phospholipase A2, group IIA (*Pla2g2a*), rat neutrophil defensin 3 (*RatNP-3*), collagen, type I, alpha 1 (*Col1a1*), and fibronectin 1 (*Fn1*) were all induced by carcinogen treatment, but to a lower extent in rats fed either BB or SF. Changes in expression of these genes were further evaluated by *q*RT-PCR and results presented in Figure 4. In accordance with microarray data, there were significant main effects for both diet (*P* < 0.05) and carcinogen (*P* < 0.05) treatment for each gene examined. Generally, expression was lowest in bean-fed rats but increased in all diets with carcinogen injections. Rats injected with AOM and fed the control (AIN) diet had the highest overall expression level coinciding with the higher tumor incidence observed in these animals. Somatostatin 2 receptor was also influenced by both diet and carcinogen treatment, being basally higher in bean-fed animals and decreasing in all groups following carcinogen administration (Table 4).

## 4. Discussion

The focus of the current research was to identify potential cellular and molecular events underlying suppression of tumorigenesis by beans using a highly relevant animal model of colon cancer. We profiled global changes in gene expression affected by AOM treatment in normal-appearing colon mucosa to determine early events permissive for tumor formation and whether these changes could be attenuated by dietary treatment. Although not a primary focus of this study, tumor incidence was also assessed. As previously demonstrated, both BB- [32] and SF-fed [30, 31, 43] rats developed significantly fewer tumors overall, confirming that these diets inhibit experimental colon carcinogenesis. In the study by Hangen and Bennink [32], however, black and navy bean- fed-rats ate less and as a result had significantly lower body weights at termination of the study. Because of the inverse association between energy restriction and tumorigenesis, the tryptophan and methionine content in the black bean diets was adjusted to raise the amino acid score comparable to that of the AIN and SF diets. As a result, no significant differences in final weight gain were detected, indicating that black beans inhibit tumorigenesis by a mechanism other than energy restriction.

We found that AOM treatment most notably affected genes involved in innate defense and immunity. For example, the antimicrobial genes lysozyme, group IIA phospholipase A_2_ (*Pla2g2a; *sPLA_2_), and neutrophil (NP) defensin 3 (*RatNP-3*) were approximately 2-fold higher than in saline-injected controls. Several major histocompatibility class (MHC) II-associated antigens as well as *CD74*, the class II MHC-associated invariant chain, were also induced in the colon of AOM-injected rats. Epithelial cells, activated dendritic cells, and/or macrophages underlying the intestinal cell layer can function as antigen-presenting cells [44–48], and specific upregulation of these genes implies that carcinogen treatment alters immune responsiveness to luminal and/or bacterial antigens. Additionally, CD74, aside from its classical antigen transporting role, has also been reported to bind macrophage inhibitory factor (MIF), leading to nuclear factor kappa B (NF*κ*B) activation, inhibition of p53 phosphorylation, and cell proliferation [49, 50]. Another cluster of genes influenced by AOM treatment involved moderate induction of several ribosomal proteins. RNA and protein synthesis decrease as cells terminally differentiate [51], and enhanced presence of ribosomal protein transcripts is consistent with findings from other studies in the colon of rats susceptible to PhIP-induced colon cancer [52] and in animals during aging [53].

Chronic inflammation creates an environment permissive to carcinogenesis through enhanced production of lipid mediators, cytokines, and chemokines that influence cell proliferation and apoptosis [54–58]. Additionally generation of reactive oxygen and nitrogen species either directly or through activation of phagocytic cells can lead to oxidative damage of DNA [55, 59, 60]. The mucosal barrier, comprised of goblet cell-derived mucin and reinforced by tight junctions normally protects the epithelium and limits activation of immune cells within the lamina propria [61–64]. Dysfunctions in one or more components of the mucosal barrier have been implicated in the pathogenesis of inflammatory bowel diseases (IBD) [65–67] as well as in CRC [68–72]. For example, increased intestinal permeability and altered structure of tight junction proteins precedes relapse in individuals with IBD [73]. Additionally, Soler et al. [70] noted defects in tight-junction permeability in normal mucosa and in colon tumors from carcinogen-treated animals, and the surface epithelial cells of aberrant crypt foci are reported to be deficient in mature goblet cells, have altered mucin composition, and contain irregular microvilli [74]. Alterations in one or more components of the mucosal barrier would support our findings of higher immune responsive genes observed in carcinogen treated animals and is likely an early and permissive event in promotion of colon carcinogenesis.

Because both black beans and soy flour reduced tumorigenesis to a similar extent in this study, we next evaluated genes that were either co-induced or suppressed compared to AIN-fed animals. We identified several transcripts associated with proliferation and apoptosis to be regulated in a diet-dependent manner. An interesting finding was a 2-fold higher expression of *Ceacam1* in rats fed either BB or SF. *Ceacam1* encodes a cell-surface glycoprotein expressed in the differentiated cell compartment of colonic crypts and expression correlates positively with normal rates of apoptosis [75, 76]. A tumor suppressive function for *Ceacam1* has been suggested due to loss of expression in hyperplastic polyps, adenomas, as well as in human colon cancers that precedes defects in the *APC* pathway [75]. Somatostatin receptor 2 (*Sst2*), which mediates anti-proliferative responses to the hormone somatostatin [77, 78], was also more abundant in bean-fed animals and levels tended to decrease following AOM treatment. These results are consistent with decreased expression of *sst2* in human colon tumors [79] and with findings from Xiao et al. [80] who demonstrated enhanced colonic mRNA and serum protein levels of somatostatin in rats fed either whey or soy protein isolate. Bean-fed rats also exhibited a lower expression of the mitotic genes* Ccnb1*, *Cdc2*, and *Top2a*. *Top2a* is involved in a variety of processes including DNA replication, chromosome segregation, and maintenance of chromosome structure [81]. Protein expression has been detected primarily in the actively proliferating cells at the base of the crypt and levels increase during tumorigenesis [82]. Binding of cyclin B1 to Cdc2 is required for cells to enter mitosis at the G2 checkpoint [83, 84]. Although these genes can be regulated at the transcriptional level in a p53-dependent manner [84], the fold change differences between diets would more likely suggest an increase in the proportion of cells undergoing terminal differentiation. This is further supported by the anatomical distribution of Topoisomerase II alpha and CEACAM1 along the crypt-lumen axis and implies a general effect of bean-feeding on maintaining normal crypt cell homeostasis.

Other clusters of genes similarly affected by bean-feeding highlight differences in fiber sources on colon cell physiology. For example, the most highly induced class of genes affected by bean diets included those involved in water channel and ion transport (*Aqp8, Scnn1g, Slc12a7, Tfrc, Slc5a1, Slc16a1*) and energy metabolism (*Hmgcs2, Aldob, Pck1, Hadhb, Ech1*). These changes are consistent with the physiological effects of fermentable fibers on intestinal function. Bacterial fermentation of dietary fibers and resistant starch produces the short-chain fatty acids (SCFA), acetate, propionate, and butyrate [85, 86]. SCFAs are trophic to the normal colonic epithelium, enhance water and sodium absorption, increase mucosal blood flow, and modulate enterohormone release [87, 88]. Butyrate in particular is an important energy source for colonocytes and can induce growth arrest, differentiation, and/or apoptosis of colon epithelial cells *in vitro* [89, 90]. Augenlicht et al. [90, 91] demonstrated that butyrate metabolism, through mitochondrial *β*-oxidation, is important for induction of apoptosis both *in vitro* and *in vivo*. Dsyregulation of genes involved in energy metabolism, such as *Hmgcs2,* have been observed in inflammatory conditions of the colon [92] and during tumorigenesis [42, 93], suggesting a potential link between enhanced colonic expression and reduced cancer risk.

Despite the large number of genes influenced by carcinogen or diet alone, only 5 transcripts were significantly affected by both treatments. It was originally hypothesized that gene changes within this group would be the most important to understanding dietary modulation of tumorigenesis, with particular interest in those that paralleled tumor incidence data. We identified that transcripts for antimicrobial genes (*Pla2g2a*, *RatNP-3*) and extracellular matrix components (*Col1a1*, *Fn1*) were induced by AOM treatment in all diets, but to a much lesser extent in bean-fed animals, whereas somatostatin receptor 2 (*Sstr2*) showed the opposite trend. sPLA_2_ (*Pla2g2a*) and NP defensin 3 (*RatNP-3)* exhibit antimicrobial activity and together with other proteins play an important role in mucosal epithelial defense [94–100]. sPLA_2_ is a multifunctional protein induced in a variety of inflammatory and neoplastic conditions [101–107]. Enhanced expression has been reported in colon tumors of patients with familial adenomatous polyposis [108], in areas adjacent to sporadic colon tumors [109], in inflammatory bowel disease [101, 104, 105, 110], as well as in carcinogen-induced tumors in rodents [41, 111], suggesting a promoting role in colonic neoplasia. Aside from antimicrobial activity [95], sPLA_2_ may coordinate immune defenses by enhancing neutrophil function [112] and contributing to eicosanoid synthesis [103, 113, 114]. Similar to sPLA_2_, NP defensin 3 exhibits antimicrobial activity as well as other immune modulatory roles [99, 115, 116]. Normal colonic expression of alpha defensins is low [96, 97], but expression is induced during inflammation [96, 97, 117, 118], potentially by the proinflammatory cytokines IL-1*β*, TNF-*α*, and IL-6 [119]. Recently, increased levels of human neutrophil (HNP) defensins 1–3 were identified in individuals with colorectal tumors [120–122] as well as in the serum and colon from patients with active IBD [117], indicating that alpha defensins may be a plausible and early biomarker for gastrointestinal disease. Dietary modulation of these genes in addition to a lower expression of other innate immune genes, including toll-like receptor 4 (*Tlr4*) and *Dmbt1*, further suggest bean feeding inhibits tumor promotion by limiting microbially induced inflammation.

Alterations in extracellular matrix (ECM) components are commonly observed during inflammation and carcinogenesis [123–126] and contribute to number of processes including cell adhesion and migration, wound healing, angiogenesis, and immune cell migration and activation [123, 127]. Additionally, changes in the distribution and expression of different ECM proteins have also been reported along the crypt axis [128–130] suggesting a role in normal epithelial migration and differentiation [130–132]. *Col1a1* expression is upregulated in colon cancer and in other hyperproliferative disorders [133], but normal colonic expression has not been previously reported. Fibronectin has been identified as a downstream target of the Wnt/*β*-catenin pathway, which is frequently altered in colorectal carcinogenesis [134, 135]. Additionally, Kolachala et al. [136] reported expression is localized to surface epithelial cells and protein levels increase during the active and restitution phase of dextran sodium-induced colitis in mice. This was associated with induction of the *α*5-integrin receptor, increased cell attachment, and activation of the NF*κ*B signaling pathway. Although important in restitution to injury, several bacteria also contain binding sites for fibronectin, and increased apical secretion may influence adherence to mucosal surfaces, thereby potentiating inflammation [137–140]. The lower expression of these genes in bean-fed animals treated with carcinogen may represent fewer preneoplastic lesions and/or a more general effect of diet on maintaining mucosal integrity.

Our findings provide a strong basis for future studies on legumes and colon cancer prevention, however, there are a few limitations to the current study. First, although microarrays are a powerful tool for biomedical research, one limitation to this technology is decreased sensitivity for the detection of low-abundance genes [141]. Additionally, differences in gene expression may not directly reflect changes in protein levels nor account for other posttranslational modifications such as protein phosphorylation that may also be involved in dietary modulation of colon cancer [141]. Additional studies should be undertaken to address the functional involvement of gene changes and corresponding proteins from this study to further confirm postulated roles in chemoprevention. Another potential limitation was the use of colonic mucosal scrapings rather than whole colon tissue. Mucosal scrapings yield a heterogeneous cell population consisting primarily of colonocytes, with lesser amounts of intraepithelial lymphocytes, macrophages, and endothelial cells. Although cancer evolves from genetic and epigenetic alterations arising in epithelial cells, there is increasing recognition for the importance of the microenvironment in the carcinogenic process [142]. For example, infiltrating immune cells in the lamina propria may contribute to tumor development through generation of reactive oxygen species [143, 144] as well as local production of cytokines, chemokines, and other lipid mediators which can influence carcinogenesis by promoting angiogenesis and disrupting cell cycle regulation [142]. Although our primary interests were in epithelial gene changes, gene expression patterns in other cell types were likely underrepresented and should be considered in future studies to more fully understand the impact of legumes on colon cancer development.

## 5. Conclusions

In summary, dietary habits are strongly associated with colon cancer risk, and this research lends further support to epidemiological and experimental data that consumption of bean-based diets inhibits colon cancer development. The finding that beans reduce markers of colonic inflammation is consistent with the inverse association of long-term non-steroidal anti-inflammatory drug (NSAID) use on CRC risk [145, 146]. Further, it is well recognized that individuals previously treated for colon cancer are at a higher risk of recurrence of the disease [147]. This observation is proposed to be related to molecular abnormalities in areas surrounding cancer tissue [148, 149]. Results from this study suggest that some abnormalities may be related to changes in cytokinetics, the innate immune system, and extracellular matrix components. We speculate dietary modulation of these genes is associated with reduced inflammation, possibly through enhancing mucosal barrier function. Confirming some of these gene changes in humans, further identifying what causes these changes to occur and determining if bean consumption can reverse these changes would strengthen the relationship of bean consumption on colon cancer inhibition and may provide useful adjunct therapy for those at risk.

## Supplementary Material

The sequences of the primer pairs used to confirm gene changes with *q*RT-PCR. Gene specific primers for cell division cycle 2 (*Cdc2*), cyclin B1 (*Ccnb1*), topoisomerase II alpha (*Top2A*), group IIA secretory phospholipase A2 (*Pla2g2a*), fibronectin 1 (*Fn1)*, collagen, type I, alpha 1 (*Col1a1*), rat neutrophil (NP) defensin 3 (*RatNP-3*), aquaporin 8 (*Aqp8*), and 3-hydroxy-3-methylglutaryl-Coenzyme A synthase 2 (*Hmgcs2*) were designed with the Primer Express 2.0 program (Applied Biosystems, Foster City, CA). *β*-actin was used as an internal control.

## Figures and Tables

**Table 1:** Nutrient composition of experimental diets^1^.

Ingredient	g/100 g diet		
	AIN	Black bean	Soy flour
Casein	20	2.7	—
Black bean flour	—	74	—
Defatted soy flour	—	—	34
Cornstarch	45	—	36
Sucrose	1.8	1.8	1.8
Total lipid^2^	17	17	17
Total fiber in diet^3^	11	11	11
Mineral mix	3.9	3.9	3.9
Vitamin mix	1.1	1.1	1.1
Methionine	0.33	0.40	0.33
Tryptophan	—	0.004	—
Calcium carbonate	0.25	0.25	0.25
Choline bitartrate	0.28	0.28	0.28
Tert-butylhydroquinone	0.002	0.002	0.002

^1^Nutrient compositions were calculated from the USDA nutrient database and Reeves [38].

^2^Total lipid content in diets calculated based on natural occurring lipids and added fat. The SFA : MUFA : PUFA composition of all diets was 1 : 1.2 : 1.

^3^Total fiber content (11.25%) in all diets based on the amount present from individual dietary components as well as added fiber (cellulose).

**Table 2:** Select genes significantly affected by carcinogen (AOM) treatment in the distal colonic epithelium of male F344 rats^1^.

Gene symbol	Gene title	AOM (fold change)
(I) Extracellular matrix, cell adhesion, cytoskeleton		

*Fn1*	fibronectin 1	1.7
*Col1a1*	collagen, type I, alpha 1	1.6
*Vim*	vimentin	1.5
*Tmsb10*	thymosin, beta 10	1.3
*Col3a1*	collagen, type III, alpha 1	1.3

(II) Immune, defense, inflammation, stress		

*Pla2g2a*	phospholipase A2, group IIA (platelets, synovial fluid)	2.4
*Cd74*	Cd74 molecule, major histocompatibility complex, class II invariant chain	2.1
*Lyz2*	lysozyme 2	2.1
*RT1-Db1*	RT1 class II, locus Db1	2.0
*RT1-DMb*	RT1 class II, locus DMb	1.7
*RatNP-3*	defensin RatNP-3 precursor	1.7
*RT1-Da*	histocompatibility 2, class II antigen E alpha	1.6
*RT1-Ba*	RT1 class II, locus Ba	1.5
*Cxcl13*	chemokine (C-X-C motif) ligand 13	1.5
*RT1-Bb*	RT1 class II, locus Bb	1.5
*Ccl2*	chemokine (C-C motif) ligand 2	1.4
*Mif*	macrophage migration inhibitory factor	1.3
*RT1-M3-1*	RT1 class Ib, locus M3, gene 1	1.3
*Irf7*	interferon regulatory factor 7	1.3
*Cxcr4*	chemokine (C-X-C motif) receptor 4	1.3
*Il15*	interleukin 15	0.6

(III) Protein processing, synthesis, degradation		

*Rps7*	ribosomal protein S7	1.4
*Rps15*	ribosomal protein S15	1.4
*Pfdn2*	prefoldin subunit 2	1.4
*Hspe1*	heat shock protein 1 (chaperonin 10)	1.3
*Rpl37*	ribosomal protein L37	1.3
*Rpl4*	ribosomal protein L4	1.3
*Psmb4*	proteasome (prosome, macropain) subunit, beta type 4	1.3
*Rpl3l*	ribosomal protein L3-like	1.3
* Rps4x*	ribosomal protein S4, X-linked	1.3
*Rps17*	ribosomal protein S17	1.3
*Rpl36al*	ribosomal protein L36a-like	1.3
*Rps9*	ribosomal protein S9	1.3

^1^Data expressed as mean-fold change normalized to saline-injected animals (*n* = 12/group). All genes presented were significantly altered compared to saline-injected animals (*P* < 0.05).

**Table 3:** Genes similarly affected by bean-feeding (BB and SF) in the distal colonic epithelium of male F344 rats^1^.

Gene symbol	Gene title	BB	SF
(I) Cell cycle, cell growth and maintenance, apoptosis			

*Ceacam1*	carcinoembryonic antigen-related cell adhesion molecule 1	2.3*	1.9*
*Rb1*	retinoblastoma 1	2.2*	2.2*
*Gadd45a*	growth arrest and DNA-damage-inducible, alpha	1.5*	1.5*
*Bax*	Bcl2-associated X protein	1.3	1.4*
*Egln3*	EGL nine homolog 3 (C. elegans)	0.67*	0.79
*Wfdc1*	WAP four-disulfide core domain 1	0.67*	0.50*
*Ccnb1*	cyclin B1	0.66*	0.85
*Rfc4*	replication factor C (activator 1) 4	0.59*	1.1
*Cdc2*	cell division cycle 2, G1 to S and G2 to M	0.58*	0.83
*Top2a*	topoisomerase (DNA) II alpha	0.57*	0.85
*Bub1b*	budding uninhibited by benzimidazoles 1 homolog, beta (S. cerevisiae)	0.49*	0.60*

(II) Channel, transporters, & carriers			

*Aqp8*	aquaporin 8	2.7*	2.9*
*Scnn1g*	sodium channel, nonvoltage-gated 1 gamma	2.2*	2.0*
*Apoa1*	apolipoprotein A-I	1.6	2.4*
*Slc12a7*	solute carrier family 12 (potassium/chloride transporters), member 7	1.6*	1.4*
*Slc5a1*	solute carrier family 5 (sodium/glucose cotransporter), member 1	1.5*	1.3
*Slc16a1*	solute carrier family 16, member 1 (monocarboxylic acid transporter 1)	1.5*	1.5*
*Atp1a1*	ATPase, Na^+^/K^+^ transporting, alpha 1 polypeptide	1.4*	1.3*
*Lgals9*	lectin, galactoside-binding, soluble, 9	1.3*	1.3*
*Kcnk1*	potassium channel, subfamily K, member 1	1.3*	1.3*
*Slco1a1*	solute carrier organic anion transporter family, member 1a1	0.68	0.35*
*SLC16A6*	solute carrier family 16, member 6 (monocarboxylic acid transporter 7)	0.61*	0.82
*Fabp5*	fatty acid binding protein 5, epidermal	0.48*	0.64*
*Ttpa*	tocopherol (alpha) transfer protein	0.47*	0.66*

(III) Electron transport, oxidoreductase, detoxification			

*Cyp4b1*	cytochrome P450, family 4, subfamily b, polypeptide 1	3.6*	2.2*
*Gstm5*	glutathione S-transferase, mu 5	1.5*	1.5*
*Gstm1*	glutathione S-transferase mu 1	1.4*	1.3
*Cyp27a1*	cytochrome P450, family 27, subfamily a, polypeptide 1	1.4*	1.5*
*Prdx6*	peroxiredoxin 6	1.4	1.5*
*Cyp2d1 *	cytochrome P450, family 2, subfamily d, polypeptide 1	1.4*	1.4*
*Por*	P450 (cytochrome) oxidoreductase	1.4*	1.4*
*Cyb5a*	cytochrome b5 type A (microsomal)	1.4*	1.3*
*Tst*	thiosulfate sulfurtransferase, mitochondrial	1.3*	1.3*

(IV) Energy metabolism			

*Hmgcs2*	3-hydroxy-3-methylglutaryl-Coenzyme A synthase 2	2.2*	2.1*
*Aldob*	aldolase B, fructose-bisphosphate	2.1*	1.8*
*Pck1*	phosphoenolpyruvate carboxykinase 1 (soluble)	2.0*	1.8*
*Ech1*	enoyl coenzyme A hydratase 1, peroxisomal	1.6*	1.6*
*Glul*	glutamate-ammonia ligase	1.4*	1.2*
*Hadhb*	hydroxyacyl-Coenzyme A dehydrogenase/3-ketoacyl-Coenzyme A thiolase/enoyl-Coenzyme A hydratase (trifunctional protein), beta subunit	1.3*	1.3*
*Cbr1*	carbonyl reductase 1	1.2	1.5*
*Hadha*	hydroxyacyl-Coenzyme A dehydrogenase/3-ketoacyl-Coenzyme A thiolase/enoyl-Coenzyme A hydratase (trifunctional protein), alpha subunit	1.2*	1.3*
*Pfkp*	phosphofructokinase, platelet	0.67*	0.76*
*Gpd2*	glycerol-3-phosphate dehydrogenase 2, mitochondrial	0.67*	0.82
*Acsl4*	acyl-CoA synthetase long-chain family member 4	0.64*	0.80
*Pyy*	peptide YY	0.64*	0.72*
*Gcg*	glucagon	0.57*	0.81
*Scd*	stearoyl-CoA desaturase (delta-9-desaturase)	0.53*	0.72
*Scd1*	stearoyl-Coenzyme A desaturase 1	0.47*	0.65

(V) Extracellular matrix, cell adhesion, cytoskeleton			

*Sdc1*	syndecan 1	1.3*	1.3*
*Sparc*	secreted protein, acidic, cysteine-rich (osteonectin)	1.0	0.48*
*Fn1*	fibronectin 1	0.66*	0.61*
*Tubb5*	tubulin, beta 5	0.65*	0.92
*Col1a1*	collagen, type I, alpha 1	0.53*	0.52*

(VI) Immune, defense, inflammation, stress			

*Hspa1a*	heat shock 70 kD protein 1A	1.9*	1.4
*RT1-EC2*	RT1 class Ib, locus Aw2	1.5*	1.4*
*Tlr4*	toll-like receptor 4	0.78*	0.68*
*Mif*	macrophage migration inhibitory factor	0.71*	0.88
*Dmbt1*	deleted in malignant brain tumors 1	0.64*	0.74*
*Pla2g2a*	phospholipase A2, group IIA (platelets, synovial fluid)	0.50*	0.39*
*RatNP-3*	defensin RatNP-3 precursor	0.48*	0.69*
*Cxcl14*	chemokine (C-X-C motif) ligand 14	0.41*	0.48*

(VII) Nucleic acid binding, transcription regulation			

*Nr1d2*	nuclear receptor subfamily 1, group D, member 2	2.2*	1.3
*Nfib*	nuclear factor I/B	1.5*	1.4*
*Vdr*	vitamin D (1,25- dihydroxyvitamin D3) receptor	1.4	1.6*
*Syt4*	synaptotagmin IV	0.82	0.65*
*Egr2*	early growth response 2	0.77*	0.60*
*Nr4a2*	nuclear receptor subfamily 4, group A, member 2	0.70*	0.75*
*Egr1*	early growth response 1	0.50*	0.66*

(VIII) Signal transduction			

*Gchfr*	GTP cyclohydrolase I feedback regulator	1.9*	1.8*
*Ppm1b*	protein phosphatase 1B, magnesium dependent, beta isoform	1.6*	1.4
*Mapk14*	mitogen activated protein kinase 14	1.3*	1.4*
*Gucy2c*	guanylate cyclase 2C	1.3*	1.4*
*P2ry2*	purinergic receptor P2Y, G-protein coupled 2	0.81	0.61*
*Ptpn3*	protein tyrosine phosphatase, non-receptor type 3	0.79*	0.73*
*P2ry6*	pyrimidinergic receptor P2Y, G-protein coupled, 6	0.69*	0.68*
*Pld1*	phospholipase D1	0.68*	0.85
*Ptpn18*	protein tyrosine phosphatase, non-receptor type 18	0.63*	0.83*
*Ptpro*	protein tyrosine phosphatase, receptor type, O	0.62*	0.72*
*Fzd1*	frizzled homolog 1 (Drosophila)	0.62*	0.74*

^1^Data expressed as mean-fold change normalized to the AIN group (*n* = 8/group). *denotes significant difference compared to the control (AIN) diet (*P* < 0.05).

**Table 4:** Genes significantly affected both by carcinogen (AOM) and dietary treatment in the distal colonic epithelium of male F344 rats^1^.

Gene symbol	Gene title	Saline-treated			AOM-treated		
		AIN	BB	SF	AIN	BB	SF
*Pla2g2a*	phospholipase A2, group IIA	1.0	0.64	0.33	2.5	0.97	1.2
*RatNP-3*	NP defensin 3	1.0	0.44	0.70	1.7	0.78	1.0
*Col1a1*	collagen, type I, alpha 1	1.0	0.67	0.59	2.3	0.94	0.97
*Fn1*	fibronectin 1	1.0	0.72	0.77	2.0	1.2	1.0
*Sstr2*	somatostatin receptor 2	1.0	1.4	1.7	0.69	1.1	1.2

^1^Data expressed as mean fold-change differences standardized to the AIN (saline-injected) group (*n* = 4/group). There were significant main effects for injection type (saline versus AOM) and dietary treatment (AIN versus BB or SF) for each gene listed (*P* < 0.05).
