# Supplementary material for: Microarray Analyses of Genes Differentially Expressed by Diet (Black Beans and Soy Flour) during Azoxymethane-Induced Colon Carcinogenesis in Rats
Source: J Nutr Metab. 2012 Feb 8;2012:351796. doi: 10.1155/2012/351796 (PMC3306975; doi:10.1155/2012/351796)
Supplement: Supplementary file 1 — The sequences of the primer pairs used to confirm gene changes with qRT-PCR. Gene specific primers for cell division cycle 2 (Cdc2), cyclin B1 (Ccnb1), topoisomerase II alpha (Top2A), group IIA secretory phospholipase A2 (Pla2g2a), fibronectin 1 (Fn1), collagen, type I, alpha 1 (Col1a1), rat neutrophil (NP) defensin 3 (RatNP-3), aquaporin 8 (Aqp8), and 3-hydroxy-3-methylglutaryl-Coenzyme A synthase 2 (Hmgcs2) were designed with the Primer Express 2.0 program (Applied Biosystems, Foster City, CA). β-actin was used as an internal control. [file 351796.f1.docx]

| Supplementary Table 1. Primer pairs used for *q*RT-PCR^1^ | | |
| --- | --- | --- |
| Gene | Forward Primer (5´-3´) | Reverse Primer (5´-3´) |
| *β-Actin* | AAC CGT GAA AAG ATG ACC CAG AT | CAC AGC CTG GAT GGC TAC GT |
| *Cdc2* | GCT TGG ACT TGC TCT CGA AAA | ATG GGT GCT TAA GGG CCA TT |
| *Ccnb1* | TGT GCA CCT GCC GAA GAA | ACC ATC GTC TGC ATC TAC ATT CAT C |
| *Top2a* | GTC CGT TGA AAG AAT CTA TCA GAA AA | CCA CAG AGC CGA TGT AGG TAT CT |
| *Pla2g2a* | GCC TGA TCT TTC CCC AAC ACT | CTA CGC AGC AGG AAG TTG GAT |
| *RatNP-3* | CTC CCT GCA TAC GCC AAA | AAC AGA GTC GGT AGA TGC G |
| *Col1a1* | AGG AGA GAG TGC CAA CTC CA | GTG CTT TGG AAA ATG GTG CT |
| *Fn1* | TGT GAT TTG GTC TGG GAT CA | GTG TTT GGA CAC AGC CAC AG |
| *Hmgcs2* | TGC CCA AAC GTC TAG ACT CC | GGA GAG AAG TTC ACC TTG TGG T |
| *Aqp8* | TGG AAC CTG GAA CTC CTT TG | GAG GGC TGG AAA AAT GAA CA |
| ^1^ *Abbreviations:* *Cdc2*, cell division cycle 2; *Ccnb1*, cyclin B1; *Top2a*, topoisomerase II alpha; *Hmgcs2,* 3-hydroxy-3-methylglutaryl-Coenzyme A synthase 2; *Aqp8,* Aquaporin 8; *Pla2g2a,* phospholipase A2, group IIA; *RatNP-3*, rat neutrophil defensin 3; *Col1a1*, collagen, type I, alpha 1; *Fn1*, fibronectin 1. | | |
